# Editorial

**DOI:** 10.1515/medgen-2024-2050

**Published:** 2024-12-03

**Authors:** Johannes Zschocke, Fleur S. van Dijk

**Affiliations:** Medical University Innsbruck Institute of Human Genetics, Department of Genetics Peter-Mayr-Str. 1 6020 Innsbruck Austria; London North West University Healthcare NHS Trust National EDS service Harrow United Kingdom

The classification of (genetic) disorders is often a hotly debated topic among scientists, medical professionals and, increasingly, affected individuals and their families. This is certainly the case with Ehlers-Danlos syndromes (EDS).

Interestingly, EDS began as a singular entity at the beginning of the 20th century. However, by the middle of the 20th century, it became clear that there were distinct groups of patients with specific clinical features that followed Mendelian inheritance patterns. This soon led to the Berlin nosology of 1986, followed by the Villefranche classification of 1997, until in 2017 13 types of EDS were reported, 12 of which were monogenic conditions.

The variable but overlapping core features of generalised joint hypermobility, skin hyperextensibility and connective tissue fragility, and their frequent occurrence in the general population, have made it difficult to decide how to define EDS and which conditions should be included under the term. Over the years there have been discussions about moving from numerical EDS types to descriptive terms, which was finally agreed in the Villefranche classification when, for example, EDS type IV was renamed vascular EDS (vEDS). Nevertheless, it seems that for many health professionals and affected individuals “EDS” is still considered to be a single condition, with patients saying “they have EDS” and health professionals referring to a patient as “having EDS”.

The most likely reason for this is that the majority of people with “EDS” do not have a monogenic condition, but have hypermobile EDS (or Hypermobility Spectrum Disorder, HSD), conditions characterised by generalised joint hypermobility and other clinical features, but for which no underlying genetic cause has yet been identified. The monogenic types of EDS are a smaller group by comparison.

Unfortunately, referring to patients as having “EDS” has led to miscommunication and mismanagement for individuals with hypermobile EDS, but certainly also for individuals with monogenic EDS. This is illustrated by the tears of a young woman with vEDS as she explained how she attended the emergency department with abdominal pains, had imaging after a long wait, was sent home without the imaging results, and then was called back to the hospital because an arterial aneurysm was found.

Experiences such as these have led some people with monogenic EDS to ask whether their condition could be renamed and no longer be called “EDS”. However, this could be difficult as it would mean another major change for scientists, medical professionals, affected individuals and their families.

The solution could be much simpler and would involve health professionals and patients always defining the type of EDS they have been diagnosed with, as EDS in itself is not a diagnosis.

The importance of defining the type of EDS is emphasised by the articles published in this issue of Medizinische Genetik. The first article focuses on the clinical manifestations of different monogenic types of EDS, including many images to illustrate the spectrum of clinical features seen in these individuals. The next two articles provide information on genetic and non-genetic investigation strategies for monogenic EDS, which should assist in the efficient and effective diagnosis of affected individuals. The final article provides a clinical update on vEDS, a rare monogenic disorder characterised by generalised tissue fragility that can lead to significant complications from a young age. It is hoped that this collection of articles will assist in making more – and more accurate – diagnoses, thereby facilitating appropriate treatment and management of these often debilitating and sometimes life-threatening conditions.

